# Trends in Cardiovascular Mortality Among a Cohort of Children and Young Adults Starting Dialysis in 1995 to 2015

**DOI:** 10.1001/jamanetworkopen.2020.16197

**Published:** 2020-09-09

**Authors:** Elaine Ku, Charles E. McCulloch, Patrick Ahearn, Barbara A. Grimes, Mark M. Mitsnefes

**Affiliations:** 1 Division of Nephrology, Department of Medicine, University of California, San Francisco; 2 Division of Pediatric Nephrology, Department of Pediatrics, University of California, San Francisco; 3 Department of Epidemiology and Biostatistics, University of California, San Francisco; 4 Division of Nephrology, Department of Medicine, Stanford University, Stanford, California; 5 Division of Pediatric Nephrology, Department of Pediatrics, Cincinnati Children’s Hospital, Cincinnati, Ohio

## Abstract

**Importance:**

Survival of patients receiving dialysis has improved during the last 2 decades. However, few studies have examined temporal trends in the attributed causes of death (especially cardiovascular-related) in young populations.

**Objective:**

To determine temporal trends and risk of cause-specific mortality (ie, cardiovascular and infectious) for children and young adults receiving dialysis.

**Design, Setting, and Participants:**

This retrospective cohort study examined the records of children and young adults (aged <30 years) starting dialysis between 1995 and 2015 according to the United States Renal Data System database. Analyses were performed between June 2019 and June 2020. Fine-Gray models were used to examine trends in risk of different cardiovascular-related deaths. Models were adjusted for age, sex, race, neighborhood income, cause of end-stage kidney disease, insurance type, and comorbidities. Analyses were performed separately for children (ie, age <18 years) and young adults (between ages 18 and 30 years). Follow-up was censored at death or administratively, and transplantation was treated as a competing event.

**Exposures:**

Calendar year.

**Main Outcomes and Measures:**

Cardiovascular cause-specific mortality.

**Results:**

A total of 80 189 individuals (median [interquartile range] age, 24 [19-28] years; 36 259 [45.2%] female, 29 508 [36.8%] Black, and 15 516 [19.3%] Hispanic white) started dialysis and 16 179 experienced death during a median (interquartile range) of 14.3 (14.0-14.7) years of follow-up. Overall, 40.2% of deaths were from cardiovascular-related causes (6505 of 16 179 patients). In adjusted analysis, risk of cardiovascular-related death was stable initially but became statistically significantly lower after 2006 (vs 1995) in those starting dialysis as either children (subhazard ratio [SHR], 0.74; 95% CI, 0.55-1.00) or adults (SHR, 0.90; 95% CI, 0.83-0.98). Risk of sudden cardiac death improved steadily for all age groups, but to a greater degree in children (SHR, 0.31; 95% CI, 0.20-0.47) vs young adults (SHR, 0.64; 95% CI, 0.56-0.73) comparing 2015 vs 1995. Risk of stroke became statistically significantly lower around 2010 (vs 1995) for children (SHR, 0.40; 95% CI, 0.18-0.88) and young adults (SHR, 0.76; 95% CI, 0.59-0.99).

**Conclusions and Relevance:**

In this study, the risk of cardiovascular-related death declined for children and young adults starting dialysis during the last 2 decades, but trends differed depending on age at dialysis initiation and the specific cause of death. Additional studies are needed to improve risk of cardiovascular disease in young populations.

## Key Points

**Question:**

Has the risk of death from cardiovascular disease improved in children and young adults starting dialysis during the last 2 decades?

**Findings:**

In this cohort study including 80 189 children and young adults initiating dialysis over a 20-year period, risk of cardiovascular-related mortality improved overall for both children and young adults over time. The risk of sudden cardiac death improved more for children than adults, while improvements in stroke-related mortality were slower to occur and less pronounced.

**Meaning:**

Risk of cardiovascular-related mortality in a young population receiving dialysis improved overall, but further studies are needed to understand variations in changes to the risk of different cardiovascular-related causes of death.

## Teaser

This cohort study examines trends in cardiovascular mortality in children and young adults initiating dialysis between 1995 and 2015.

## Introduction

The life expectancy of a patient who develops end-stage kidney disease (ESKD) during childhood is known to be significantly diminished compared with children in the general population.^1^ Young adults treated with dialysis have also been noted to have mortality rates that are more than 100-fold higher than their age-matched counterparts in the general population.^1,2^ The leading cause of death among children and adults treated with dialysis has been attributed to cardiovascular disease (CVD) and, in particular, sudden cardiac death (SCD).^2,3^

While long-term mortality risk among children treated with dialysis has improved during the last 2 decades,^4,5^ the exact reasons for these improvements have remained unclear. Potential possibilities include improvements in cardiovascular risk factor modification or infection prevention over time in the dialysis population.

The objective of this study was to compare trends over time in mortality from CVD-related causes of death among a cohort of children and young adults younger than 30 years of age who started dialysis between 1995 and 2015. We compared changes in CVD-related mortality risk with infection-related causes of death. We also compared risk factors for CVD-related causes of death in those starting dialysis as children vs as young adults.

## Methods

### Study Population and Data Source

We performed a retrospective cohort study of children and young adults younger than 30 years of age who started maintenance dialysis between January 1995 and December 2015 using data from the United States Renal Data System (USRDS), the national ESKD registry.^6^ We included individuals as old as 30 years at time of dialysis initiation because prior studies have used this upper limit when focusing on young adult populations.^7^

Patient demographic characteristics (ie, age at incident ESKD, sex, and race), cause of ESKD (ie, diabetes, hypertension, glomerulonephritis, cystic, or other), zip code, and date of ESKD onset were abstracted from the Centers for Medicare & Medicaid Services 2728 (CMS-2728) Medical Evidence (MEDEVID) Form and Patients file in the USRDS. Zip code was used to determine the median household income of patients’ neighborhoods using values from the American Community Survey between 2006 and 2010, which encompasses data from the midpoint of the follow-up period included in our study.^8^ Initial ESKD treatment modality (transplantation vs dialysis) was determined at the first service date as listed in the MEDEVID file. The University of California, San Francisco institutional review board considered this study exempt human participants research due to the deidentified nature of the publicly available data. Our study adhered to the Strengthening the Reporting of Observational Studies in Epidemiology (STROBE) reporting guideline for cohort studies.

### Primary Outcomes

We abstracted death dates and primary causes of death (overall, CVD-related, and infection-related mortality) from the USRDS Patients file through December 2015. In addition, we also subtyped CVD-related causes of death into deaths attributed to SCD or arrhythmias (henceforth referred to as SCD), myocardial infarction (MI), stroke, or heart failure and/or cardiomyopathy. Cause of death was abstracted from the CMS death notification form that is submitted to the USRDS. If the exact cause of death was missing but date of death was known, we classified these deaths as other causes of death.

### Patient Characteristics at Dialysis Initiation

We first determined the demographic characteristics of patients at the start of dialysis in the overall cohort and among those who died. Because we were interested in differences in risk factors for different causes of death in those starting dialysis as children vs adults, we compared characteristics of children vs adults separately at time of dialysis initiation. We defined children as patients younger than 18 years and young adults as patients aged between 18 and 30 years to adhere to the current National Institutes of Health definition of children.^9^ Next, to determine whether differences in patient characteristics at the start of dialysis may account for any potential differences observed in mortality rates, we examined the demographic and comorbid patient characteristics reported on the CMS-2728 form at time of dialysis initiation in 5-year intervals between 1995 and 2015.

### Statistical Analysis

We determined the rate of death (per 100 person-years) for the overall cohort. Death rates were also determined by 10-year (as opposed to 5-year) intervals separately for children vs adults.

#### Temporal Trends in Risk of Death

We began analysis of temporal trends in all-cause and cause-specific mortality using 1995 as our reference year and thus included an incident cohort of children and young adults treated with dialysis. We examined temporal trends in risk of CVD-related, infection-related, or all-cause death using Fine-Gray models (a survival analysis approach that allows for the consideration of potential competing risks, such as transplantation or other causes of death outside the specific outcome of interest). We determined the first transplantation date using the USRDS Patients file. Time in these Fine-Gray models was measured in years from dialysis initiation until death. The primary variable was calendar year, and the outcome was cause-specific (or all-cause) death. We included both linear and quadratic terms for calendar year to account for potential nonlinearities in temporal trends in the risk of death in all models. These models were adjusted for demographic factors (ie, age at ESKD onset, sex, race, cause of ESKD, median neighborhood income by patient zip code) and comorbidities (ie, coronary artery disease, diabetes, heart failure, and stroke as reported on the CMS-2728 form) that were present at time of dialysis initiation. We considered these models our primary models.

All models were stratified by whether the individual started dialysis as a child or young adult. The relative subhazard ratio (SHR) was plotted for the risk of the different causes of death by calendar year, using 1995 as the reference year. In sensitivity analysis, we repeated our primary models using Cox models adjusted for the same covariates as described above, but treated transplantation as a censoring (rather than a competing) event, and models were repeated for all-cause, CV-related, and infectious-related causes of death.

Next, we repeated our Fine-Gray models for CVD-specific causes of death (SCD, MI, stroke, and heart failure and/or cardiomyopathy as separate outcomes) and treated other causes of death (besides the outcome of interest) and transplantation as competing risks in separate models for children and young adults. To determine whether the risk of dying from any specific cause was potentially affected by changes in the proportion of missing causes of death over time, we compared the percentage of deaths that were missing by 5-year calendar intervals, both for the overall cohort and separately in children and young adults.

#### Risk Factors for CVD-Related Mortality

Finally, we examined the risk of CVD-related death based on covariates of interest using Fine-Gray models stratified by decade of dialysis initiation (1995-2004 vs 2005-2010) to capture potential changes in risk factors over time. In these models, the quadratic term for calendar year was excluded if it did not achieve statistical significance. All analyses were performed using SAS version 9.0 (SAS Institute). *P *values <.05 were considered statistically significant. All analyses were completed between June 2019 and June 2020.

## Results

### Study Population

We included 80 189 children and young adults in this study. Median (interquartile range [IQR]) age of the cohort was 24 (19-28) years, 36 259 participants (45.2%) were women and girls, 29 508 (36.8%) were Black individuals, and 15 516 (19.3%) were Hispanic white individuals (Table 1). The median (IQR) follow-up period in this cohort was 14.3 (14.0-14.7) years, and the most common cause of ESKD in this cohort was glomerulonephritis (37.4%) (Table 1). The most common cause of death was CVD (6505 of 16 179 [40.2%]) followed by infection-related causes (2332 [14.4%]).

**Table 1.** Characteristics of the Overall Cohort at Time of Dialysis Initiation

Characteristic	Patients, No. (%)					
	Overall (N = 80 189)	Children (n = 15 398)	Young adult (n = 64 791)	Died (n = 16 179)	Died of CV cause (n = 6505)	Died of infectious cause (n = 2332)
Age at dialysis initiation, y						
Mean (SD)	22.5 (7.2)	10.3 (5.8)	25.3 (3.5)	24.3 (6.4)	24.7 (5.8)	24.2 (6.8)
Median (IQR)	24 (19-28)	12 (5-15)	26 (23-28)	26 (22-29)	26 (23-29)	26 (23-29)
Girls and young women	36 259 (45.2)	6942 (45.1)	29 317 (45.2)	8328 (51.5)	3354 (51.6)	1217 (52.2)
Race/ethnicity						
Non-Hispanic White	29 533 (36.8)	6600 (42.9)	22 933 (35.4)	5034 (31.1)	2016 (31.0)	577 (24.7)
Black	29 508 (36.8)	3994 (25.9)	25 514 (39.4)	8458 (52.3)	3391 (52.1)	1369 (58.7)
Asian	3833 (4.8)	605 (3.9)	3228 (5.0)	407 (2.5)	178 (2.7)	65 (2.8)
Hispanic White	15 516 (19.3)	3727 (24.2)	11 789 (18.2)	1841 (11.4)	750 (11.5)	256 (11.0)
Other	1799 (2.2)	472 (3.1)	1327 (2.0)	439 (2.7)	170 (2.6)	65 (2.8)
Income, median (IQR), $	45 346 (35 966-58 304)	47 228 (37 411-61 861)	44 827 (35 714-57 620)	41 144 (32 958-52 402)	41 194 (32 960-52 219)	40 726 (32 744-52 945)
Attributed cause of ESKD						
GN	30 003 (37.4)	5646 (36.7)	24 357 (37.6)	4493 (27.8)	1830 (28.1)	621 (26.6)
Cystic or urologic	3710 (4.6)	1364 (8.9)	2346 (3.6)	537 (3.3)	176 (2.7)	108 (4.6)
Hypertension	13 092 (16.3)	498 (3.2)	12 594 (19.4)	2445 (15.1)	1086 (16.7)	263 (11.3)
Diabetes	11 940 (14.9)	101 (0.7)	11 839 (18.3)	4562 (28.2)	2167 (33.3)	486 (20.8)
Other	21 444 (26.7)	7789 (50.5)	13 655 (21.1)	4142 (25.6)	1246 (19.2)	854 (36.6)
Comorbidities at ESKD onset						
CAD	881 (1.1)	51 (0.3)	830 (1.3)	420 (2.6)	224 (3.4)	43 (1.8)
CHF	5331 (6.6)	318 (2.1)	5013 (7.7)	2055 (12.7)	944 (14.5)	261 (11.2)
Stroke	1053 (1.3)	131 (0.9)	922 (1.4)	423 (2.6)	181 (2.8)	62 (2.7)
Hypertension	53 811 (67.1)	6149 (39.9)	47 662 (73.6)	10 975 (67.8)	4728 (72.7)	1370 (58.7)
Diabetes	14 310 (17.8)	268 (1.7)	14 042 (21.7)	5313 (32.8)	2475 (38.0)	588 (25.2)

Abbreviations: CAD, coronary artery disease; CHF, congestive heart failure; CV, cardiovascular; ESKD, end-stage kidney disease; GN, glomerulonephritis; IQR, interquartile range.

Compared with the overall cohort, patients who died were older at time of dialysis initiation (mean [SD] age, 24.3 [6.4] years vs 22.5 [7.2] years) and more likely to be women and girls (8328 [51.5%]) or Black individuals (8458 [52.3%]) (Table 1). Patients who died were also more likely to have diabetes and less likely to have glomerulonephritis as their attributed cause of ESKD (diabetes: 4562 [28.2%] vs 11 940 [14.9%]; glomerulonephritis: 4493 [27.8%] vs 30 003 [37.4%]). Presence of diabetes or heart failure at time of dialysis initiation was more common in those who died compared with the overall cohort (diabetes: 5313 [32.8%] vs 14 310 [17.8%]; heart failure: 2055 [12.7%] vs 5331 [6.6%]) (Table 1).

Over time, the age of dialysis initiation remained similar across sequential 5-year intervals included for analysis (eTable in the Supplement). However, over time a smaller proportion of the patients starting dialysis were Black patients. In general, the baseline presence of comorbid conditions reported at time of dialysis initiation, such as heart failure or stroke, were low in this young population and not substantially different over time, with the exception of an increased prevalence of hypertension (eTable in the Supplement). Glomerulonephritis as a cause of ESKD decreased over time.

### Rates of Death and Temporal Trends in Mortality Risk

The overall death rate and rate of death attributed to CVD-related causes was higher in those who started dialysis as young adults vs as children (Table 2). In contrast, the rate of death attributed to infection-related complications was similar in children and young adults (Table 2). Cause of death was missing for 148 children (9.5%) and 1113 adults (7.6%).

**Table 2.** Death Rates by Cause-Specific Mortality in Children vs Adults

Interval	Death rate per 100 person-years (95% CI)						
	Overall	CV	Infection-related cause	SCD	MI	Stroke	CHF
**1995-2015**							
Total outcomes, No.	16 179	6505	2332	4648	511	724	440
Total population	5.1 (5.0-5.2)	2.0 (2.0-2.1)	0.7 (0.7-0.8)	1.5 (1.4-1.5)	0.2 (0.1-0.2)	0.2 (0.2-0.2)	0.1 (0.1-0.2)
Young adult	5.2 (5.2-5.3)	2.1 (2.1-2.2)	0.7 (0.7-0.8)	1.5 (1.5-1.6)	0.2 (0.2-0.2)	0.2 (0.2-0.3)	0.1 (0.1-0.2)
Child	4.0 (3.8-4.2)	1.3 (1.2-1.4)	0.7 (0.6-0.7)	0.9 (0.8-1.0)	0.08 (0.06-0.1)	0.2 (0.1-0.2)	0.1 (0.1-0.2)
**1995-2004**							
Total outcomes, No.	9861	3973	1678	2725	354	469	282
Total population	5.5 (5.4-5.6)	2.2 (2.2-2.3)	0.9 (0.9-1.0)	1.5 (1.5-1.6)	0.2 (0.2-0.2)	0.3 (0.2-0.3)	0.2 (0.1-0.2)
Children	4.4 (4.1-4.7)	1.5 (1.3-1.7)	0.8 (0.7-0.9)	1.0 (0.9-1.1)	0.1 (0.1-0.1)	0.2 (0.2-0.3)	0.1 (0.1-0.2)
Young adult	5.7 (5.6-5.8)	2.3 (2.2-2.4)	1.0 (0.9-1.0)	1.6 (1.5-1.7)	0.2 (0.2-0.2)	0.3 (0.2-0.3)	0.2 (0.1-0.2)
**2005-2015**							
Total outcomes, No.	6318	2532	654	1923	157	255	158
Total population	4.5 (4.4-4.7)	1.8 (1.8-1.9)	0.5 (0.4-0.5)	1.4 (1.3-1.4)	0.1 (0.1-0.1)	0.2 (0.2-0.2)	0.1 (0.1-0.1)
Child	3.5 (3.2-3.8)	1.1 (0.9-1.2)	0.4 (0.4-0.6)	0.7 (0.6-0.9)	0.1 (0.0-0.1)	0.1 (0.1-0.2)	0.1 (0.1-0.2)
Young adult	4.7 (4.6-4.8)	1.9 (1.8-2.0)	0.5 (0.4-0.5)	1.5 (1.4-1.5)	0.1 (0.1-0.1)	0.2 (0.2-0.2)	0.1 (0.1-0.1)

Abbreviations: CHF, congestive heart failure; CV, cardiovascular; MI, myocardial infarction; SCD, sudden cardiac death.

Of the CVD-attributed causes of death, the rate of SCD was 1.5 (95% CI, 1.4-1.5) per 100 person-years and up to 10 times higher for children (0.9 [95% CI, 0.8-1.0] per 100 person-years) and young adults (1.5 [95% CI, 1.5-1.6] per 100 person-years) compared with heart failure (0.1 [95% CI, 0.1-0.2] per 100-person years) as the attributed cause of death (Table 2). Stroke was the second most common CVD-related cause of death for both children and young adults (children: 0.2 [95% CI, 0.1-0.2] per 100 person-years; young adults: 0.2 [95% CI, 0.2-0.3] per 100 person-years). MI was rare in those who started dialysis as children during the 2 decades of follow-up (0.08 [95% CI, 0.06-0.10] per 100 person-years).

**Figure 1.**
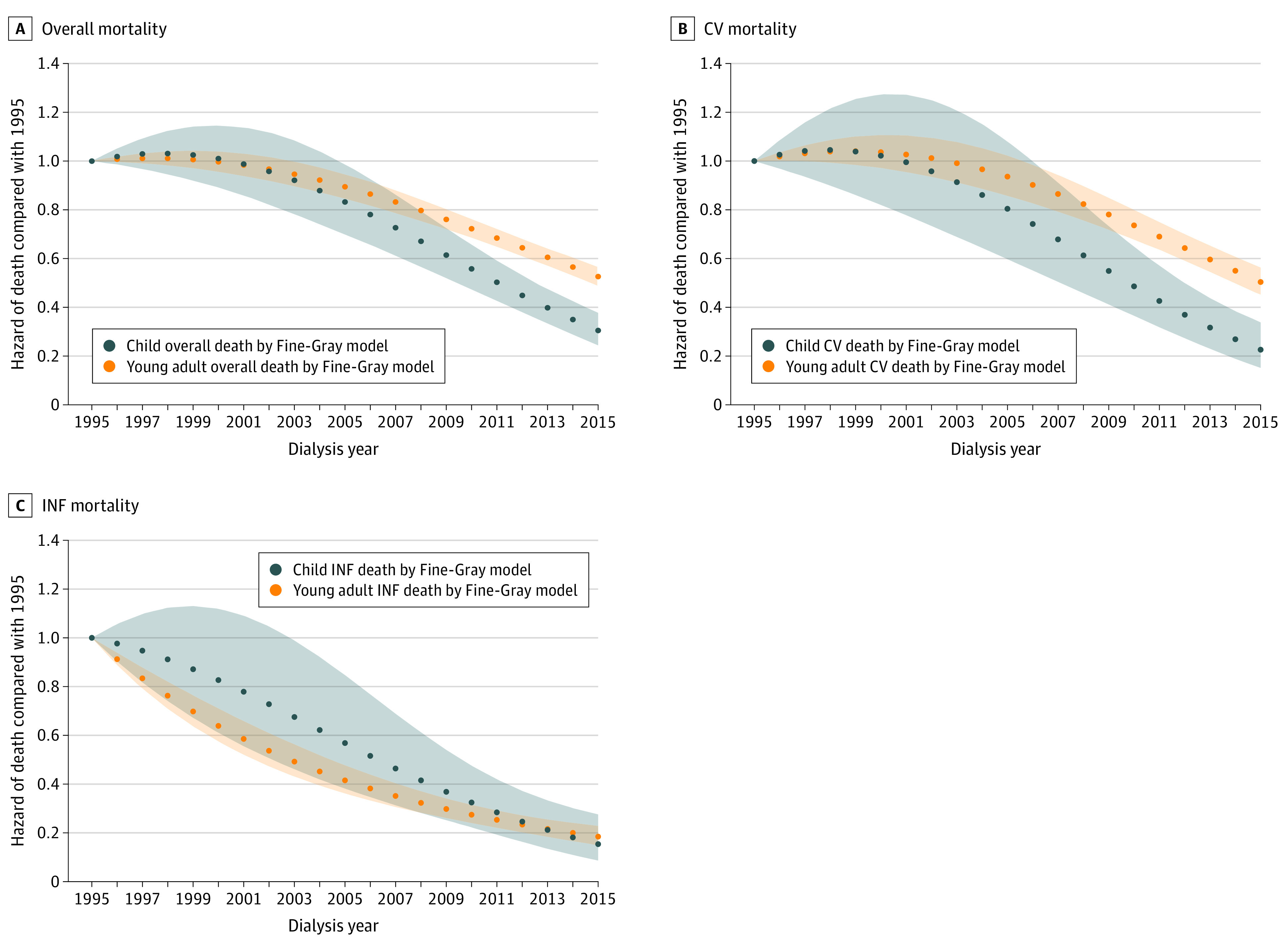
Trend in Cause-Specific Deaths Among Children and Young Adults by Calendar Year of Dialysis Initiation Using Fine-Gray Models Shaded areas represent 95% CIs for the point estimates in young adults and children. CV indicates cardiovascular; INF, infection.

When we examined trends in the overall risk of mortality by calendar year, we found that the risk of all-cause mortality did not change in linear fashion over time in those starting dialysis as children or as young adults (Wald χ^2^, 44.0; *P* < .001 for presence of nonlinearity) (Figure 1A). The risk of all-cause mortality was not statistically significantly different (compared with the reference year of 1995) until 2003 in those starting dialysis as young adults and 2005 in those starting dialysis as children, when the risk became statistically significantly lower (young adults: SHR, 0.95; 95% CI, 0.90-1.00; children: SHR, 0.83; 95% CI, 0.70-0.99; Figure 1A).

When we examined trends in CVD-related causes of death (Figure 1B and Table 2), we found that the trends were similar to those observed for all-cause mortality in children (Figure 1A) with a stable risk of CV-related death until 2006, when the risk became statistically significantly lower in those starting dialysis as children (SHR, 0.74; 95% CI, 0.55-1.00) or young adults (SHR, 0.90; 95% CI, 0.83-0.98). The risk of dying from an infection-related cause decreased steadily over time for both young adults and for children (Figure 1C and Table 2), although the risk was statistically significantly lower after 1995 for young adults (SHR, 0.91; 95% CI, 0.89-0.94) but only after 2003 for children (SHR, 0.68; 95% CI, 0.46-0.99).

**Figure 2.**
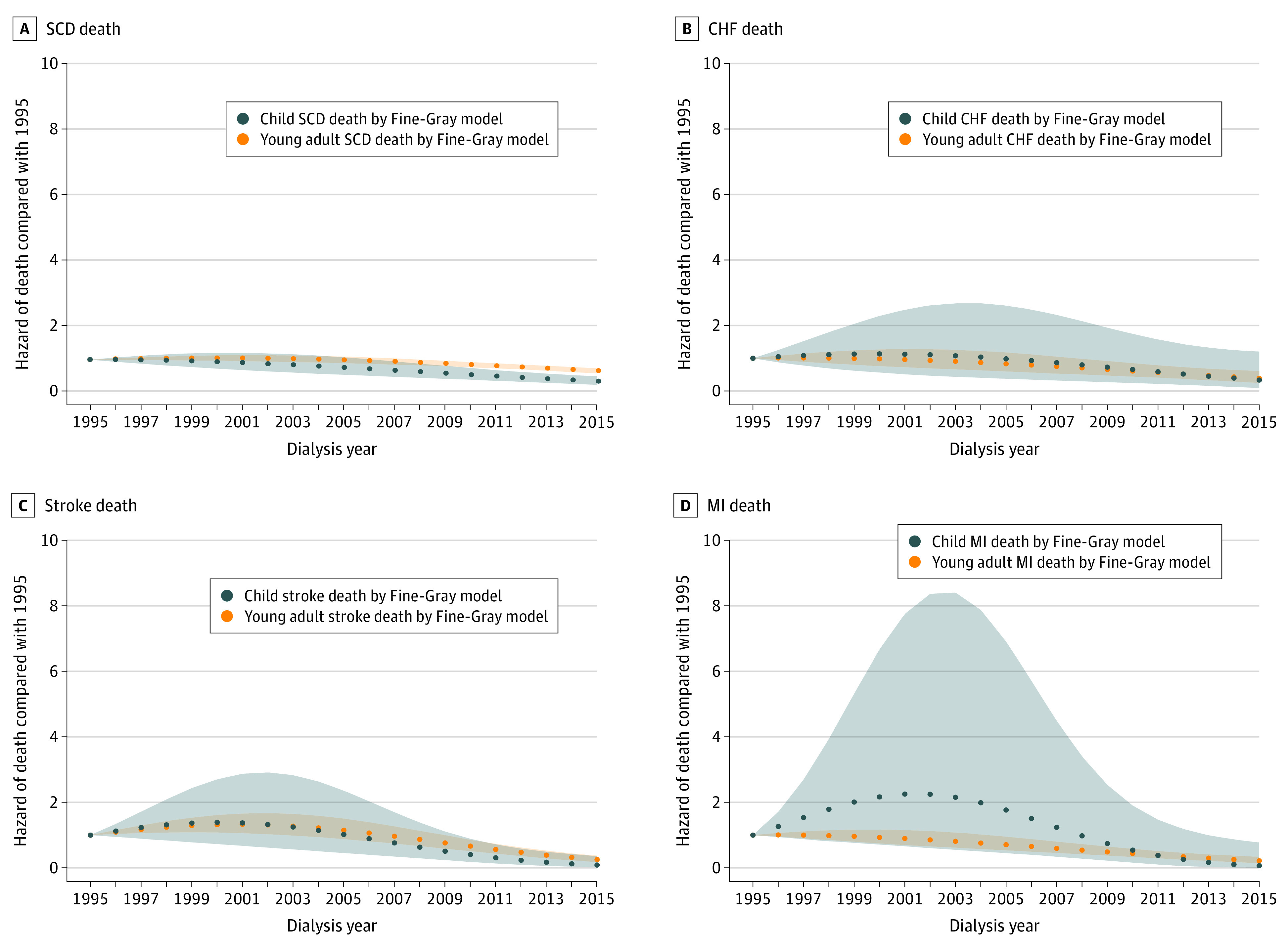
Trend in Cause-Specific Cardiovascular Disease-Related Deaths Among Children and Young Adults by Calendar Year of Dialysis Initiation Using Fine-Gray Models Shaded areas represent 95% CIs for the point estimates in young adults and children. CHF indicates congestive heart failure; MI, myocardial infarction; SCD, sudden cardiac death.

When we examined trends in cause-specific CV-related deaths over time, we found that the risk of dying of SCD was linear in children but not for young adults (children: Wald χ^2^, 2.3; *P* = .13; young adults: Wald χ^2^, 13.3; *P* < .001 for presence of nonlinearity) (Figure 2A). In contrast, trends differed for heart failure–related mortality in those starting dialysis as children vs as young adults: while the risk of dying of heart failure was not statistically significantly different in children over time, the risk began to decline for those starting dialysis as young adults after 2008 (SHR 0.70; 95% CI 0.50-0.98) (Figure 2B).

The risk of dying of an MI was statistically significantly lower after 2005 compared with 1995 for young adults (SHR, 0.70; 95% CI, 0.52-0.95), but in children, the difference in risk of dying of an MI was not statistically significantly different during most of the follow-up duration (although the event rate was very low and the confidence intervals were wide) (Figure 2C). The difference in risk of dying of a stroke was not statistically significantly different until rates improved after 2009 to 2010 for both those starting dialysis as children (SHR, 0.40; 95% CI, 0.18-0.88) and as young adults (SHR, 0.76; 95% CI, 0.59-0.99) (Figure 2D).

### Risk Factors for CV-Related Death

The median follow-up time was 19.9 (25th percentile, 17.5) years in children and 13.9 (IQR, 13.5-14.2) years in young adults. When we examined characteristics associated with the risk of death of CV-attributed causes using Fine-Gray models accounting for the competing risk of transplantation and other causes of death, we found that in those starting dialysis as children, risk of a CVD-related death was higher among those who were younger at the time of dialysis initiation (per year increase in age: SHR, 0.92; 95% CI, 0.84-0.94) in the most recent decade (Table 3). This finding differed for those starting at dialysis as young adults in the first decade of follow-up, such that older age at dialysis initiation was associated with a higher risk of CV-related mortality (per year increase in age: SHR, 1.03; 95% CI, 1.02-1.04). In both children and young adults, having Black race was a relatively consistent risk factor for CV-related mortality (Table 3). Presence of heart failure was associated with higher risk of CV-related mortality for both young adults (SHR, 1.72; 95% CI, 1.53-1.94) and children (SHR, 5.09; 95% CI, 2.82-9.17) between 2005 and 2015; similar associations were noted between 1995 and 2004 (Table 3).

**Table 3.** Association Between Characteristics at Time of Dialysis Initiation and Cardiovascular Disease–Related Causes of Death in Children vs Young Adults by Decade of Follow-up

Risk factor	Young adults, SHR (95% CI)		Children, SHR (95% CI)	
	1995-2004	2005-2015	1995-2004	2005-2015
Age at dialysis initiation per 1-y increase	1.03 (1.02-1.04)	1.01 (0.99-1.02)	1.00 (0.97-1.02)	0.92 (0.89-0.94)
Female vs male patients	1.17 (1.09-1.25)	1.23 (1.13-1.34)	1.25 (1.00-1.56)	1.08 (0.80-1.47)
Race				
Non-Hispanic White	1 [Reference]	1 [Reference]	1 [Reference]	1 [Reference]
Black	1.51 (1.39-1.64)	1.30 (1.18-1.44)	1.85 (1.43-2.39)	1.38 (0.95-2.01)
Hispanic White	0.76 (0.67-0.86)	0.78 (0.68-0.89)	0.63 (0.44-0.91)	0.85 (0.56-1.29)
Asian	0.92 (0.74-1.14)	0.70 (0.55-0.91)	0.65 (0.28-1.50)	1.22 (0.56-2.68)
Median income	1.00 (1.00-1.00)	1.00 (1.00-1.00)	1.00 (1.00-1.00)	1.00 (1.00-1.00)
Cause of ESKD vs cystic disease				
GN	1.00 (0.81-1.25)	1.11 (0.82-1.51)	1.03 (0.68-1.56)	0.98 (0.54-1.79)
Hypertension	1.19 (0.95-1.50)	1.14 (0.84-1.56)	1.83 (1.01-3.31)	2.55 (1.27-5.15)
Diabetes	1.64 (1.24-2.16)	1.47 (1.05-2.05)	1.08 (0.30-3.86)	1.11 (0.31-4.01)
Comorbidities at ESKD onset				
CAD	1.58 (1.30-1.91)	1.39 (1.08-1.80)	7.81 (3.55-17.2)	1.85 (0.74-4.65)
CHF	1.54 (1.39-1.70)	1.72 (1.53-1.94)	3.37 (2.28-4.98)	5.09 (2.82-9.17)
Stroke	1.64 (1.32-2.05)	1.53 (1.20-1.94)	1.94 (0.84-4.44)	0.94 (0.30-2.91)
Hypertension	0.91 (0.84-0.99)	1.00 (0.90-1.12)	1.09 (0.86-1.38)	1.39 (0.98-1.97)
Diabetes	1.55 (1.29-1.86)	2.16 (1.83-2.55)	1.53 (0.65-3.55)	2.81 (1.38-5.70)
Calendar year per 1-y increase	1.00 (0.99-1.01)	0.94 (0.92-0.95)	0.97 (0.93-1.01)	0.86 (0.81-0.90)

Abbreviations: CAD, coronary artery disease; CHF, congestive heart failure; ESKD, end-stage kidney disease; GN, glomerulonephritis; SHR, sub-hazard ratio.

## Discussion

This study is among the largest studies that have examined temporal trends in the causes of death among young adults and children who have started dialysis, analyzing an incident cohort of more than 80 000 children and young adults who started dialysis during the last 2 decades. CVD remains the most common attributed cause of death in children and young adults treated with dialysis, although the overall risk of mortality from CV-related causes declined significantly after 2006 compared with the reference year for both cohorts. While overall CV mortality risk has declined, the trends differed depending on the age at dialysis initiation and the specific cause of death.

To our knowledge, our study is among the first to compare and contrast temporal trends and cause-specific mortality risk in a cohort of children vs young adults starting dialysis. Traditionally, young adults have been analyzed together with older adults and excluded from pediatric studies,^5,10^ even though the cause of ESKD in young adults and their disease trajectory may have many parallels to childhood disease. This juxtaposition reveals a number of important observations: although death rates are higher among young adults treated with dialysis, the median time of follow-up was only 7 years longer for those with onset of their disease in childhood compared with young adulthood. It is likely that children who remain on dialysis for prolonged periods may have had greater severity of illness (and were potentially ineligible for kidney transplantation) and that contributed to the cause of death. However, children have overall better access to transplantation than adults, and hence their average duration of dialysis should be shorter than that of young adults prior to transplantation.^11,12^ Given these considerations, the fact that the all-cause mortality rate among children remaining on dialysis was almost as high as that of young adults belies the impression that pediatric patients receiving dialysis may have better survival on dialysis than young adults.

Disappointingly, despite advances in our knowledge surrounding the burden of CV disease in a young population receiving dialysis,^5^ the relative improvements in the risk of CV-related mortality observed for young adults starting dialysis was less than that for children starting dialysis. Whether this is because of differences in the pathophysiology of the nature of the CV events in children vs young adults treated with dialysis or differential recognition of CVD risk by adult practitioners (who may be less attuned to the CV risk of young individuals despite their ESKD status) is unclear. It is especially concerning that Black patients starting dialysis are at higher risk than patients from other racial and ethnic groups for CV-related mortality.

The improvement in risk of CV causes of death was attributable largely to steady improvements in the mortality rates for SCD or arrhythmias, which together remain the most common cause of death (as in prior studies).^3,13^ The reasons for the improvement in risk of SCD or arrhythmias are unclear. Although SCD and arrhythmias may be less amenable to traditional risk modification, such as treatment of dyslipidemia or hypertension, and potentially more related to nontraditional CV risk factors, such as anemia or volume overload, we nevertheless observed that this risk has declined over time for children more so than for young adults.^14,15,16,17^

Stroke was the second leading cause of CVD-related deaths in both children and young adults, which we believe to be an important finding. Stroke prevalence has been reported to be increasing in the general young adult population as well and remains associated with significant morbidity and mortality.^18,19^ Whether the stroke-related deaths among children and young adults with ESKD were associated with uncontrolled hypertension vs other factors remains unclear. However, in general, the risk of stroke appears to have steadily improved in the most recent 5-year interval within our follow-up period, which contrasts with observations in the general population.^20,21^

### Strengths and Limitations

The strengths of our analyses include the large sample size, large number of events, and the inclusion of both children and young adults for study. Our study is also among the first to examine cause-specific mortality in children and young adults starting dialysis. However, there are a number of limitations to this study. First, we note that cause of death was missing in approximately 8% of patients, and misclassification of causes of death may have occurred, which would be more likely to bias our results toward the null. We also acknowledge that median income values by zip code may not have remained constant throughout the follow-up period, although we did use values derived from the midway point in our follow-up period. While the USRDS uniformly captures all ESKD and death events while patients remain in the US, we were unable to account for patients who may have emigrated from the US. Furthermore, we lack more granular data that may be needed to determine the exact reasons for changes in temporal trends in mortality over time, or changes in treatment of cardiovascular risk factors.

## Conclusions

In conclusion, although mortality rates have improved overall in a cohort of children and young adults starting dialysis during the last 2 decades, for some outcomes risk actually increased initially only to then improve more recently, and trends varied depending on whether individuals started dialysis as children vs as young adults. Given that CVD remains the most common cause of death in this population, strategies to further improve the CVD risk profile in this young population are needed to enhance survival, and modification of nontraditional CV risk factors may be needed to ensure continued improvements in outcomes for young populations starting on dialysis.

## Notes

Supplement. **eTable 1.** Differences in characteristics over time of patients starting dialysis by calendar period (1995-2005 vs. 2006-2015) **eFigure 1.** Cohort Derivation **eFigure 2.** Trends in Rates of Death by Year
