# Supplementary material for: Trends in Cardiovascular Mortality Among a Cohort of Children and Young Adults Starting Dialysis in 1995 to 2015
Source: JAMA Netw Open. 2020 Sep 9;3(9):e2016197. doi: 10.1001/jamanetworkopen.2020.16197 (PMC7489869; doi:10.1001/jamanetworkopen.2020.16197)
Supplement: Supplement. — eTable 1. Differences in characteristics over time of patients starting dialysis by calendar period (1995-2005 vs. 2006-2015) eFigure 1. Cohort Derivation eFigure 2. Trends in Rates of Death by Year [file jamanetwopen-e2016197-s001.pdf]

Supplementary Online Content

Ku E, McCulloch CE, Ahearn P, Grimes BA, Mitsnefes MM. Trends in Cardiovascular Mortality Among a Cohort of Children and Young Adults Starting Dialysis in 1995 to 2015. *JAMA Netw Open*. 2020;3(9):e2016197. doi:10.1001/jamanetworkopen.2020.16197

eTable 1. Differences in Characteristics Over Time of Patients Starting Dialysis by Calendar Period (1995-2005 vs. 2006-2015)

eFigure 1. Cohort Derivation

eFigure 2. Trends in Rates of Death by Year

This supplementary material has been provided by the authors to give readers additional information about their work.

© 2020 Ku E et al. *JAMA Network Open.*

eTable 1: Differences in characteristics over time of patients starting dialysis by calendar period (1995-2005 vs. 2006-2015)

**Descriptive Characteristics**	**1995-1999** N=18519	**2000-2004** N=18841	**2005-2009** N=19961	**2010-2015** N=22868
Mean age (yrs) ± SD at dialysis initiation	22.6 ± 7.0	22.2 ± 7.3	22.4 ± 7.2	22.6 ± 7.3
Median age (yrs) [IQR] at dialysis initiation	25 (19-28)	24 (19-28)	24 (19-28)	25 (20-28)
Female (%)	45.1	45.1	44.9	45.7
Race/ethnicity (%)				
NHW	39.2	35.5	36.4	36.4
Black	39.6	37.0	36.0	35.0
Asian	4.0	4.6	4.8	5.5
Hispanic white	14.9	20.1	20.8	21.1
Other	2.3	2.8	2.0	2.0
Median income ($) [IQR]	44460 [35213-57708]	45045 [35780-58244]	45881 [36471-58671]	45751 [36382-58487]
Attributed Cause of ESKD (%)				
GN	40.1	39.8	36.9	33.8
Cystic/urologic	5.4	4.8	4.3	4.2
Hypertension	14.9	15.2	16.2	18.5
Diabetes	15.4	14.1	14.4	15.5
Other	24.2	26.0	28.2	28.0
Co-morbidities at ESKD onset (%)				
CAD	1.2	1.3	1.1	0.9
CHF	6.7	7.1	6.7	6.2
Stroke	1.2	1.1	1.4	1.4
Hypertension	59.8	65.2	70	72.1
Diabetes	17.3	16.7	17.8	19.3

GN = glomerulonephritis; IQR = interquartile range; SD = standard deviation

© 2020 Ku E et al. *JAMA Network Open.*

eFigure 1. Cohort Derivation.

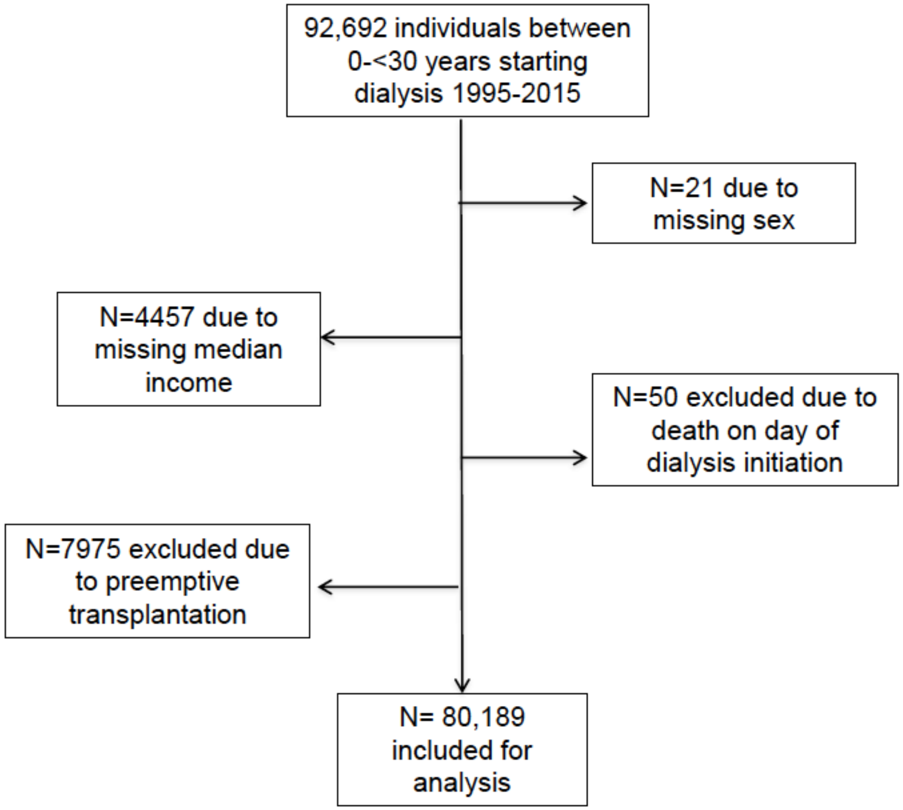

© 2020 Ku E et al. *JAMA Network Open.*

eFigure 2. Trends in Rates of Death by Year

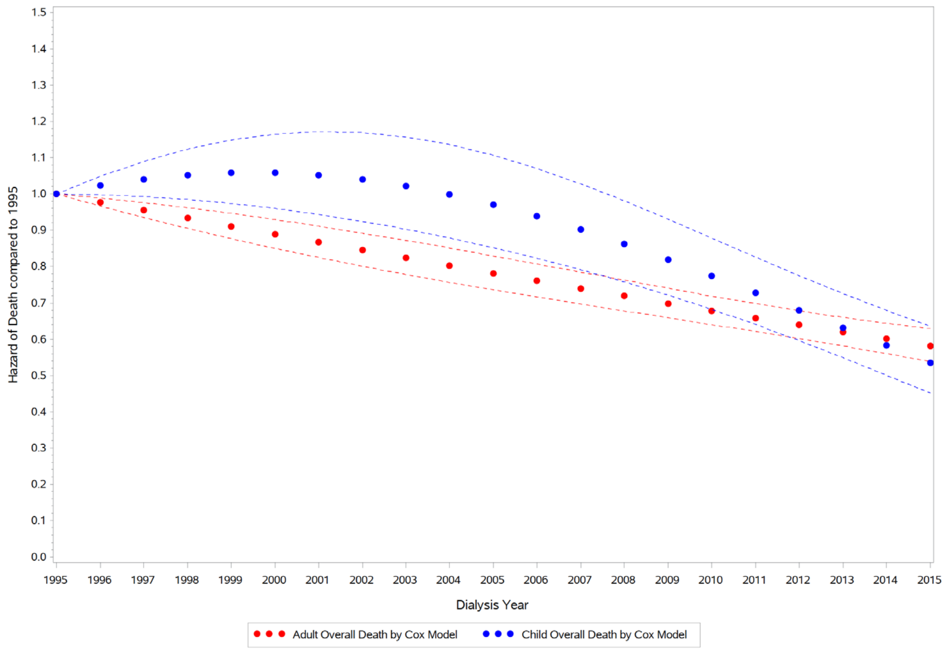

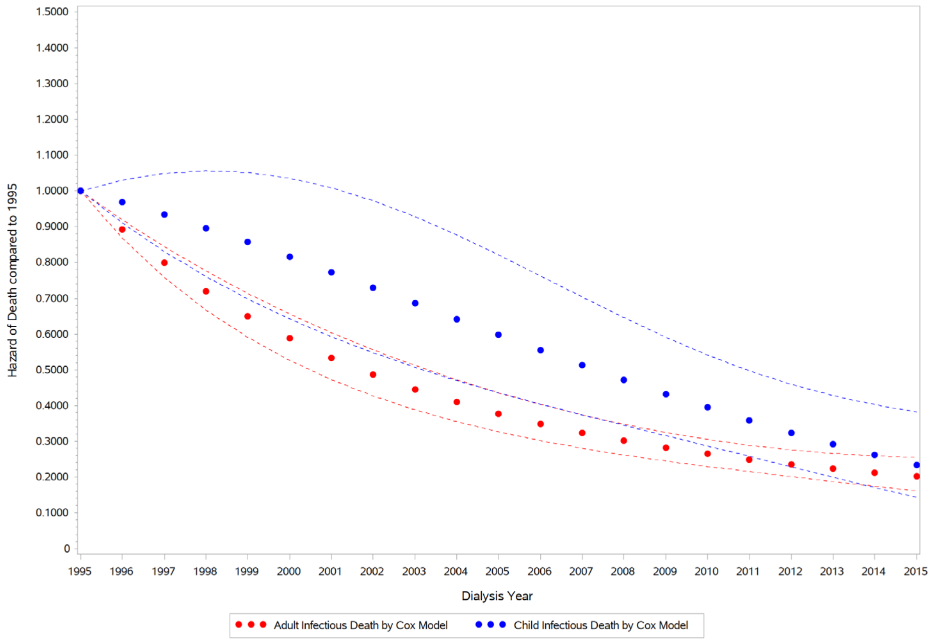

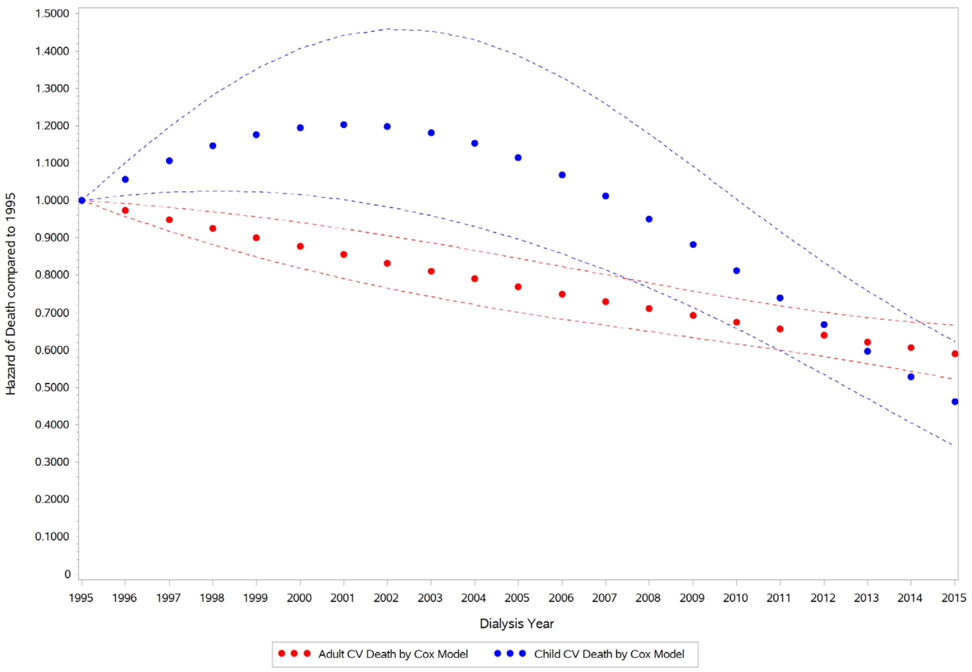

©
